# AI and Polyp Detection During Colonoscopy

**DOI:** 10.3390/cancers17050797

**Published:** 2025-02-26

**Authors:** Marco Spadaccini, Maddalena Menini, Davide Massimi, Tommy Rizkala, Roberto De Sire, Ludovico Alfarone, Antonio Capogreco, Matteo Colombo, Roberta Maselli, Alessandro Fugazza, Luca Brandaleone, Antonio Di Martino, Daryl Ramai, Alessandro Repici, Cesare Hassan

**Affiliations:** 1 Department of Biomedical Sciences, Humanitas University, Via Rita Levi Montalcini 4, Pieve Emanuele, 20090 Milan, Italy; marco.spadaccini@humanitas.it (M.S.); maddalena.menini@humanitas.it (M.M.); luca.brandaleone@humanitas.it (L.B.); cesare.hassan@hunimed.eu (C.H.); 2 Department of Gastroneterology, IRCCS Humanitas Research Hospital, Via Alessandro Manzoni 56, Rozzano, 20089 Milan, Italyroberto.desire@humanitas.it (R.D.S.);; 3 Division of Gastroenterology and Hepatology, University of Utah, Salt Lake City, UT 84112, USA

**Keywords:** artificial intelligence (AI), colonoscopy, adenoma detection rate (ADR), computer-aided detection (CADe), colorectal cancer (CRC), polyp detection

## Abstract

Colorectal cancer (CRC) prevention depends on effective colonoscopy; yet variability in adenoma detection rates (ADRs) and missed lesions remain significant hurdles. Artificial intelligence-powered computer-aided detection (CADe) systems offer promising advancements in enhancing polyp detection. This review examines the role of CADe in improving ADR and reducing adenoma miss rates (AMRs) while addressing its broader clinical implications. CADe has demonstrated consistent improvements in ADRs and AMRs; largely by detecting diminutive polyps, but shows limited efficacy in identifying advanced adenomas or sessile serrated lesions. Challenges such as operator deskilling and the need for enhanced algorithms persist. Combining CADe with adjunctive techniques has shown potential for further optimizing performance. While CADe has standardized detection quality; its long-term impact on CRC incidence and mortality remains inconclusive. Future research should focus on refining CADe technology and assessing its effectiveness in reducing the global burden of CRC.

## Simple Summary

Colorectal cancer is one of the most common and deadly cancers worldwide, and early detection is crucial for prevention. Colonoscopy is the primary method for identifying pre-cancerous polyps, but variability in detection rates among physicians can lead to missed lesions. Artificial intelligence-powered computer-aided detection (CADe) systems have been introduced to assist doctors in spotting polyps more consistently during colonoscopy. This review explores the effectiveness of CADe in improving polyp detection rates, reducing missed lesions, and addressing challenges such as reliance on AI over human skill. While CADe has demonstrated an increase in polyp detection, its impact on long-term outcomes, such as reducing colorectal cancer incidence and mortality, remains uncertain. Further advancements in AI technology and additional research are needed to optimize its role in clinical practice. By improving consistency in polyp detection, CADe has the potential to enhance the quality of colonoscopy and contribute to better cancer prevention efforts.

## 1. Introduction

Colorectal cancer (CRC) represents a significant global health concern, accounting for approximately one in ten cancer cases and deaths worldwide. It is estimated to rank third in incidence and second in mortality among all cancers. High-income countries face much higher incidence rates compared to low-income regions, reflecting lifestyle and screening differences, but mortality disparities are less pronounced due to limited healthcare access in transitioning nations. As a marker of socioeconomic development, colorectal cancer incidence rises consistently with an increasing Human Development Index (HDI) in countries undergoing economic and social transitions [1,2]. This highlights the critical need for effective prevention strategies, with the early detection of colorectal polyps being especially crucial in mitigating this growing global burden—an effort that becomes even more pressing as global economic growth continues to influence lifestyle and healthcare patterns.

It is well established that screening colonoscopy significantly reduces the 10-year risk of developing colorectal cancer compared to not undergoing screening, as demonstrated by Bretthauer et al., highlighting its critical role in early detection and prevention [3]. Indeed, a single screening colonoscopy has been shown to reduce the risk of colorectal cancer by 18%, with a risk ratio of 0.82 (95% C.I. 0.70–0.93) [3].

However, a major challenge undermines the goal of reducing colorectal cancer incidence through early polyp detection: the adenoma miss rate (AMR). A meta-analysis of over 15,000 tandem colonoscopies revealed that many polyps go undetected, with overall miss rates of 26% for adenomas and 27% for serrated polyps. Certain lesions, such as proximal advanced adenomas (14%) and flat adenomas (34%), are frequently missed, emphasizing the need for enhanced detection strategies [4].

Both the European Society of Gastrointestinal Endoscopy (ESGE) and American Society for Gastrointestinal Endoscopy (ASGE) emphasize the adenoma detection rate (ADR) as a key quality indicator for colonoscopy [5,6]. An ADR is defined as the percentage of screening colonoscopies in which at least one adenoma is identified, serving as a measurable surrogate for the effectiveness of mucosal inspection. Both societies set a minimum ADR standard of 25%, as achieving this threshold is strongly associated with a lower risk not only of interval colorectal cancer but also of advanced-stage interval cancer and fatal interval cancer [7]. Indeed, missed colorectal neoplasia is the leading cause of post-colonoscopy colorectal cancer (CRC), which occurs in about 1% of cases within 10 years after screening or surveillance colonoscopy. Research indicates that 52–57% of post-colonoscopy CRC cases result from neoplastic lesions missed during the initial procedure [8,9,10,11,12].

However, maintaining such high standards turned out to be challenging. A large community-based cohort study in which 314,872 colonoscopies were performed by 136 gastroenterologists demonstrated significant variability in adenoma detection rates (ADRs), ranging from 7.4% to 52.5%. This variability carries substantial implications: patients of physicians in the highest ADR quintile had a 48% lower risk of interval cancer compared to those in the lowest quintile. Each 1% increase in the ADR is estimated to correspond to a 3% reduction in cancer risk. These findings emphasize the critical importance of high and consistent ADRs to improve patient outcomes, while the wide variability highlights the need for strategies to standardize detection rates across endoscopists [7].

These challenges emphasize the importance of developing scalable and consistent solutions to ensure uniform quality in endoscopy across diverse clinical settings. However, the key question remains: how can this be achieved?

**Figure 1:**
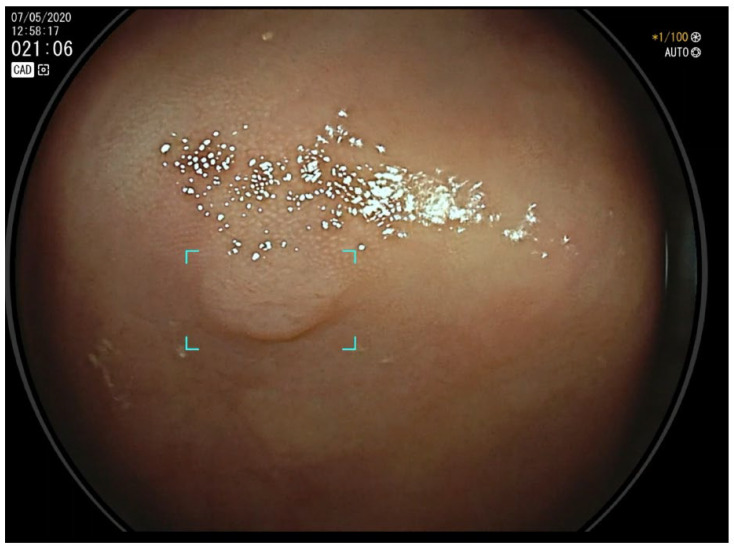
Computer-aided detection (CADe)-assisted colonoscopy. Endoscopic view of a diminutive adenoma.

Computer-aided detection (CADe) systems, a major application of artificial intelligence (AI) in gastrointestinal endoscopy, are designed to assist endoscopists during colonoscopy by identifying polyps in real time. CADe has emerged as a potential solution to address the variability in adenoma detection rates (ADRs) and to standardize the quality of colonoscopy procedures. CADe systems enhance ADRs by acting as a “second observer”, improving the detection of subtle or missed lesions and reducing reliance on the operator’s experience alone (Figure 1). By leveraging AI, CADe has the potential to mitigate human variability, raise detection consistency, and improve the overall quality of colonoscopy, making it a promising tool for standardizing care and reducing colorectal cancer incidence and mortality [13].

By presenting a concise yet comprehensive analysis, we seek to equip physicians with the knowledge needed to navigate the extensive and growing body of information on AI in colonoscopy and stay informed about its potential impact on clinical practice.

## 2. CADe and Adenoma Detection

Colorectal neoplasia detection through computer-aided detection systems has been thoroughly investigated through various randomized controlled trails (RCTs) [14,15,16,17,18,19,20].

A recent meta-analysis published in the *Annals of Internal Medicine* in 2024 by Soleymanjahi et al. confirms evidence for the effectiveness of computer-aided detection (CADe) systems in enhancing polyp detection during colonoscopy [17]. The study included 44 randomized controlled trials (RCTs) comparing standard colonoscopy with CADe-assisted colonoscopy for colonic lesion detection in patients undergoing screening, surveillance, or diagnostic procedures. Several CADe platforms were evaluated, including GI Genius (Medtronic), Fujifilm CAD EYE, EndoScreener (developed using SegNet architecture), and systems based on the You Only Look Once (YOLO) neural network. All trials utilized high-resolution colonoscopy equipment. Despite differences in CADe architectures, the systems consistently improved adenoma detection rates (ADRs), increasing from 36.7% to 44.7% (RR, 1.21; 95% CI, 1.15–1.28), and adenomas per colonoscopy (APCs), increasing from 0.78 to 0.98 (incidence rate difference, 0.22; 95% CI, 0.16–0.28). Furthermore, adenoma miss rates (AMRs) were significantly reduced in tandem colonoscopy trials, dropping from 35.3% to 16.1% (RR, 0.47; 95% CI, 0.36–0.60). Platforms based on YOLO showed a particularly notable improvement, increasing ADRs from 22% to 29% (RR, 1.36; 95% CI, 1.14–1.62), while GI Genius improved ADRs from 50% to 55% (RR, 1.16; 95% CI, 1.00–1.34), Fujifilm CAD EYE from 43% to 53% (RR, 1.21; 95% CI, 1.10–1.34), and EndoScreener from 26% to 31% (RR, 1.22; 95% CI, 1.11–1.35) [17].

These findings confirm and build upon the results of a 2023 meta-analysis by Hassan et al., which also demonstrated that CADe significantly improves ADRs, increasing it from 35.9% to 44.0% (RR, 1.24; 95% CI, 1.16 to 1.33), and reduces adenoma miss rates by 55% (RR, 0.45; 95% CI, 0.35 to 0.58) [20].

The effectiveness of CADe systems in improving adenoma detection has been further supported by focused analyses on their performance compared to standard white-light colonoscopy (WLC). A recent meta-analysis by Maida et al. compared CADe-assisted colonoscopy with WLC through the evaluation of six tandem randomized controlled trials (RCTs) involving 1718 patients. The findings revealed that CADe assistance significantly reduced the adenoma miss rate (AMR) by 54% and the polyp miss rate (PMR) by 56% compared to WLC. Importantly, the analysis exhibited low heterogeneity (18%), with all studies demonstrating consistent results, reinforcing the reliability of the findings. A sensitivity analysis focusing on screening and surveillance cases further highlighted CADe’s effectiveness in lowering miss rates for adenomas and polyps, underscoring its potential to improve the accuracy of colorectal cancer screening [21].

However, the meta-analysis by Soleymanjahi et al. highlighted significant heterogeneity in the impact of CADe on ADR [17]. This variability primarily reflected differences in effect size rather than directional inconsistency, as 40 out of 44 studies favored CADe-assisted colonoscopy. Two factors may explain this heterogeneity. First, a recent-meta-regression analysis of 23 RCTs on the effect of CADe assistance in colonoscopy showed that the substantial level of heterogeneity observed was largely attributed to differences in colonoscopy quality across studies, particularly variations in baseline ADRs in the control group and withdrawal time [22]. This is particularly important considering that the clinical context may influence CADe’s effectiveness. For instance, studies focusing on FIT-positive patients with advanced adenomas as the primary endpoint or Lynch syndrome populations did not demonstrate significant ADR improvements, highlighting limitations in these specific settings [15,23].

## 3. CADe and Colorectal Cancer

As previously mentioned, colorectal cancer is understood to arise from the gradual progression of colorectal polyps. Consequently, it is reasonable to hypothesize that improving the detection and subsequent removal of polyps could help reduce the incidence of colorectal cancer. However, the question remains: has this hypothesis been definitively demonstrated? This remains a complex issue.

Firstly, it is important to acknowledge that studies on computer-aided detection (CADe) have primarily focused on “performance metrics”, such as adenoma detection rates (ADRs), rather than patient-centered outcomes like the incidence of post-colonoscopy colorectal cancer (CRC) or mortality rates [14,15,16,17,20]. As a result, the long-term impact of CADe on overall patient health has yet to be addressed.

On the one hand, it is well established that a higher ADR is associated with a reduced risk of post-colonoscopy CRC, as undetected polyps may progress into advanced adenomas and eventually CRC [7,8,9,10,11,12]. On the other hand, it is equally important to consider that the observed increase in polyp detection may primarily involve diminutive polyps, which are less likely to evolve into CRC, rather than advanced adenomas that carry a higher risk of progression.

Supporting this, Hassan et al.’s meta-analysis revealed that CADe, compared to standard colonoscopy, did not significantly improve the mean number of advanced adenomas detected per colonoscopy [20]. Similarly, despite the optimistic results of early studies [24], Soleymanjahi et al.’s meta-analysis demonstrated that CADe has a limited impact on the number of advanced adenomas which was similar between CADe and standard colonoscopy (0.16 vs. 0.15, IRD = 0.01, 95% CI, −0.01 to 0.02) [17]. However, the meta-analysis by Soleymanjahi et al. reported a significant 16% improvement in detection rates (RR: 1.16 [95% CI: 1.02–1.32]), indicating that CADe may enhance the identification of advanced lesions.

A recent double-blind study by Sinonquel et al. addressed one of the primary limitations of previous CADe studies—operator and selection bias—while investigating CADe clinical impact. By monitoring blinded endoscopists in real time with a second observer accessing CADe, the study demonstrated that the additional ADR gain was minimal (1.1% per patient), with most additional lesions being small adenomas (<5 mm). Importantly, the detection of these lesions only altered surveillance intervals in 2.3% of cases, highlighting that the clinical relevance of CADe’s increased detection rates remains uncertain [25]. Future research should focus on long-term large trials that investigate long term clinical outcomes like CRC mortality and incidence after CADe-assisted colonoscopy.

Therefore, despite CADe’s improved ADR, its impact on long-term outcomes, such as reducing CRC incidence and mortality, remains uncertain. Addressing these gaps remains critical.

## 4. CADe and Serrated Lesions

**Figure 2:**
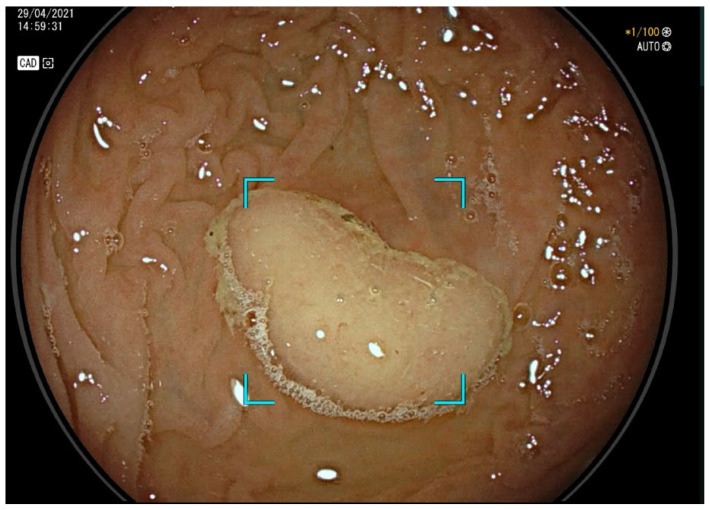
Computer-aided detection (CADe)-assisted colonoscopy. Endoscopic view of a serrated lesion.

Serrated lesions remain an highly discussed topic as they are particularly challenging to detect during colonoscopy due to their subtle appearance, flat morphology, and predilection for the proximal colon, making them more likely to be missed than other types of polyps (Figure 2) [26,27,28]. This was emphasized in a meta-analysis by Zhao et al., which found a higher miss rate for proximal advanced adenomas, serrated polyps, and flat adenomas [4]. This is even more relevant considering that research has increasingly identified serrated polyps as a significant contributor to colorectal cancer (CRC), potentially responsible for 35% of all cases, and they frequently present as interval cancers in the proximal colon [4,29,30].

Computer-aided detection (CADe) systems have demonstrated some potential in improving the detection of SSLs according to the latest meta-analyses. Soleymanjahi et al. have reported a 21% increase in SSL detection rates with CADe compared to standard colonoscopy (RR: 1.21, 95% CI, 1.04–1.42). However, the overall increase in the number of SSLs detected per colonoscopy remains modest, with an incidence rate difference (IRD) of just 0.02 (95% CI, −0.01 to 0.04) [17].

This modest improvement highlights limitations in CADe’s ability to consistently detect SSLs, especially those located in the proximal colon, where such lesions are more commonly missed. A contributing factor may be the underrepresentation of SSLs in the datasets used to train CADe algorithms, which could limit the systems’ ability to recognize the unique features of these lesions [31]. To address this limitation, future developments should focus on expanding training datasets to include a higher proportion of flat, serrated, and advanced adenomas. This will be required for future research to validate the impact of CADe on clinically significant lesions and, ultimately, colorectal cancer prevention.

## 5. CADe and Auxiliary Techniques

It could be argued that there may be other solutions to the problem of increasing and standardizing the quality, i.e., the adenoma detection rate, of colonoscopies [32]. Auxiliary techniques such as chromoendoscopy (e.g., dye-based chromoendoscopy, narrow-band imaging (NBI), and linked color imaging), and add-on device-assisted colonoscopy (i.e., Endocuff Vision) have shown efficacy in improving ADRs and reducing the adenoma miss rate (AMR), particularly for challenging lesions [33]. For instance, these methods reduced the AMR for diminutive adenomas (<5 mm) from 31% to 21% and for larger adenomas (≥10 mm) from 9% to 1%, underscoring their potential to enhance the detection of subtle or hidden lesions [7]. However, despite these improvements, these techniques alone are insufficient in resolving the persistent issue of missed adenomas and variable ADR among endoscopists [34]. Spadaccini et al. demonstrated that CADe—with an ADR 7.4% higher than HD white light endoscopy—outperformed advanced imaging techniques like chromoendoscopy (4.4% increase) and mucosal visualization systems (4.1% increase) [35]. Similarly, Miyaguchi et al. reported that linked-color imaging with artificial intelligence (LCA) significantly improved ADRs compared to LCI alone, with an ADR of 58.8% versus 43.5%, particularly for diminutive polyps (≤5 mm) [36]. These findings suggest that while auxiliary techniques offer certain benefits, the results achieved by CADe systems appear to be more substantial and reproducible. However, CADe systems are inherently limited to detecting lesions visible on mucosa adequately exposed to the camera. Therefore, increasing mucosal exposure during colonoscopy would be reasonably expected to enhance the effectiveness of CADe in detecting neoplasia. In this context, two randomized controlled trials (RCTs) have been conducted to evaluate the synergistic effects of CADe and Endocuff Vision-assisted colonoscopy (EAC) proving the efficacy of EAC in further improving the performance of endoscopists already using CADe [37,38]. The Endocuff Vision (Arc Medical Design Ltd., Leeds, UK) is a device attached to the tip of the colonoscope, featuring a circular row of eight flexible arms designed to flatten colonic folds during withdrawal without obstructing the insertion process. Multiple randomized controlled trials (RCTs) have evaluated its effectiveness, and a recent meta-analysis by Patel Harsh et al. demonstrated a significant increase in adenoma detection rates (ADRs) with Endocuff Vision compared to standard colonoscopy (49.8% vs. 45.6%; *p* = 0.02) in a pooled analysis of 5695 patients [39].

## 6. CADe and Deskilling

The integration of computer-aided detection (CADe) systems into colonoscopy, while transformative, has raised concerns about deskilling among endoscopists. Deskilling refers to the erosion of technical and diagnostic skills due to the over-reliance on automated systems. Deskilling has emerged in various medical fields after AI has been extensively and rapidly implemented across healthcare [40,41].

Recent findings suggest that the integration of AI into clinical practice may inadvertently lower the ADR during standard colonoscopy, raising concerns about a potential deskilling effect [42,43]. A recent Polish study conducted by Budzyn et al. evaluated the change in the quality of all the diagnostic colonoscopies before and after the Al implementation to assess how AI affects endoscopists behaviour [42]. The study found that adenoma detection rates (ADRs) during standard colonoscopy without AI assistance significantly decreased following the introduction of AI tools. Specifically, ADRs dropped from 28.4% (95% CI 25.29–31.56) to 22.1% (95% CI 18.95–25.26; *p* = 0.006), representing a 6.3% absolute reduction and a 22.2% relative reduction. These findings suggest a potential deskilling effect, where endoscopists’ performance declines in the absence of AI support.

These findings highlight an important concern regarding the potential deskilling effect associated with CADe systems. While one might argue that the simplest solution would be to keep CADe switched on at all times, this approach does not address the underlying issue. CADe is not intended to replace endoscopists but rather to serve as a supportive tool that enhances and helps retain their skills. However, the existence and extent of deskilling need to be more thoroughly studied to confirm and better understand its impact. Therefore, new strategies are needed to ensure that CADe systems serve as a learning aid, fostering skill development while maintaining diagnostic proficiency. Additionally, exploring innovative ways of integrating CADe as an educational and performance-enhancing tool is essential. Strategies such as periodic AI-off training sessions, structured feedback mechanisms, and AI-assisted education programs should be investigated to maintain endoscopists’ diagnostic skills. As with any new technology, the focus should be on using it responsibly and effectively to maximize its benefits while minimizing unintended consequences.

## 7. Limitations and Future Perspectives

Computer-aided detection (CADe) systems represent a significant technological advancement in colonoscopy, with the potential of improving the quality of colorectal cancer screening through enhanced adenoma detection rates (ADRs). However, critical limitations persist that may temper their clinical impact, especially regarding CRC prevention.

While CADe effectively identifies more adenomas, much of this gain is attributed to the detection of diminutive lesions, which carry a low risk of progression to malignancy. The question remains whether this enhanced detection significantly reduces CRC incidence or merely inflates the removal of clinically insignificant polyps.

Advanced adenomas, which are more strongly associated with CRC progression, appear to benefit less from CADe implementation. Flat, serrated, and locally advanced infiltrating lesions, particularly those in the proximal colon, remain a significant challenge. As noted in recent studies, these lesions probably account for a large proportion of false negatives in CADe-assisted procedures, likely due to insufficient representation in the training datasets used to develop the algorithms [44]. Addressing this gap is vital to ensuring CADe effectiveness.

An additional concern is the risk of deskilling among endoscopists. The consistent presence of CADe could lead to reduced vigilance and a decline in the technical skills required for high-quality colonoscopy. While this phenomenon warrants further investigation, it underscores the importance of viewing CADe as a complement to, rather than a replacement for, endoscopic expertise.

At the same time, it is important to recognize CADe’s democratizing potential: by narrowing the performance gap between novice and expert endoscopists, CADe could raise the baseline quality of care, particularly in settings with variable expertise. However, it remains an operator-dependent tool; adequate mucosal exposure and meticulous endoscopic techniques are prerequisites for effective lesion detection, as CADe cannot compensate for suboptimal procedural quality.

Economic considerations, while beyond the scope of this review, are unlikely to be a primary obstacle to CADe adoption. The costs associated with its implementation may be offset by the reduction in cases of advanced CRC, which require more resource-intensive treatment [45]. Thus, the focus should remain on improving CADe’s clinical effectiveness rather than its financial feasibility. However, it is important to consider that increasing the number of adenomas resected could lead to an increase in the number of surveillance colonoscopies [46].

Looking to the future, the priority must be to refine CADe algorithms to address its limitations. The training process should be standardized and datasets should be expanded to include underrepresented lesion types such as large, flat, serrated, and infiltrating polyps [47]. These enhancements would improve the system’s ability to detect frequently missed lesions that carry a risk of malignancy, ensuring that the observed increases in ADRs translate into meaningful clinical outcomes.

Furthermore, CADe systems must demonstrate their ability to reduce CRC incidence and mortality in real-world settings, requiring robust longitudinal studies to assess patient-centered outcomes beyond procedural metrics.

As previously stated, recent progress suggests that CADe is beginning to improve detection rates for more challenging lesions, but significant room for improvement remains. Flat and serrated lesions, particularly those located in the proximal colon, are disproportionately represented in post-colonoscopy CRC cases. Algorithms must evolve to target these lesions more effectively, bridging the gap between increased detection rates and tangible reductions in cancer burden.

## 8. Conclusions

In conclusion, while CADe has already transformed the landscape of colonoscopy by enhancing ADRs and standardizing procedural quality, its current limitations restrict its potential to fully address the challenges of CRC prevention. Future efforts must focus on refining the technology to better detect clinically significant lesions, mitigating the risk of potential deskilling among endoscopists, and assessing long-term impacts on patient outcomes. By addressing these challenges, CADe has the potential to solidify its role as a transformative tool in the fight against colorectal cancer, complementing and enhancing the expertise of the endoscopist rather than replacing it.

## Abbreviations

ACN	advanced colorectal neoplasia
ACN DR	advanced colorectal neoplasia detection rate
ADR	adenoma detection rate
AI	artificial intelligence
AMR	adenoma miss rate
APC	adenoma per colonoscopy
ASGE	American Society of Gastrointestinal Endoscopy
CRC	colorectal cancer
EAC	Endocuff Vision-assisted Colonoscopy
ESGE	European Society of Gastrointestinal Endoscopy
HDI	Human Development Index
LCI	linked-color imaging
NNP	nonneoplastic polyp per colonoscopy
PDR	polyp detection rate
PPC	polyp per colonoscopy
WLC	white-light colonoscopy
YOLO	You Only Look Once
